# Implementing an Individual-Centric Discharge Process across Singapore Public Hospitals

**DOI:** 10.3390/ijerph18168700

**Published:** 2021-08-17

**Authors:** Reuben Ng, Kelvin Bryan Tan

**Affiliations:** 1 Lee Kuan Yew School of Public Policy, National University of Singapore, 469C Bukit Timah Rd, Singapore 259772, Singapore; 2 Lloyd’s Register Foundation Institute for the Public Understanding of Risk, National University of Singapore, 3 Research Link, Singapore 117602, Singapore; 3 Ministry of Health, 16 College Road, Singapore 169854, Singapore; gmsncwr@nus.edu.sg

**Keywords:** predictive analytics, public healthcare, policy implementation, acute care, change management, Asia, aging policy

## Abstract

Singapore is one of the first known countries to implement an individual-centric discharge process across all public hospitals to manage frequent admissions—a perennial challenge for public healthcare, especially in an aging population. Specifically, the process provides daily lists of high-risk patients to all public hospitals for customized discharge procedures within 24 h of admission. We analyzed all public hospital admissions (*N* = 150,322) in a year. Among four models, the gradient boosting machine performed the best (AUC = 0.79) with a positive predictive value set at 70%. Interestingly, the cumulative length of stay (LOS) in the past 12 months was a stronger predictor than the number of previous admissions, as it is a better proxy for acute care utilization. Another important predictor was the “number of days from previous non-elective admission”, which is different from previous studies that included both elective and non-elective admissions. Of note, the model did not include LOS of the index admission—a key predictor in other models—since our predictive model identified frequent admitters for pre-discharge interventions during the index (current) admission. The scientific ingredients that built the model did not guarantee its successful implementation—an “art” that requires the alignment of processes, culture, human capital, and senior management sponsorship. Change management is paramount, otherwise data-driven health policies, no matter how well-intended, may not be accepted or implemented. Overall, our study demonstrated the viability of using artificial intelligence (AI) to build a near real-time nationwide prediction tool for individual-centric discharge, and the critical factors for successful implementation.

## 1. Introduction

Frequent admissions (FAs) to public hospitals are a global and perennial challenge [1], exacerbated during pandemics. COVID-19 has brought a renewed urgency to accelerate solutions that work. Admittedly, there are numerous solutions at the proof-of-concept stage; however, few are implemented at scale. There are two roadblocks: One, research funding stops at knowledge creation (e.g., publication/patent) or prototypes. There is a massive gap in funding implementation, leaving wildly successful prototypes to languish among the weeds [2]. Two, the brilliant scientists that created these wildly successful prototypes are hardly suited to carry them to implementation—the skillsets needed are diametrically different at various points of the value chain from inception to implementation. Of broader significance, the art of analytics (implementation) is as important, if not more, than the science of analytics (prototype creation). As described by managerial epidemiologists [3,4]—a sub-discipline that applies epidemiologic techniques to healthcare management—successful implementation requires artful alignment of holes across the proverbial “Swiss cheese”: The slices include processes, human capital, culture, and senior management sponsorship.

Against this background, we present one of the first-known implementations of a nationwide predictive model for individual-centric discharge across all public hospitals in Singapore. Our paper has both conceptual and practical significance. Conceptually, few studies have reported models that could be implemented prior to discharge. We advanced the field by showing the need to include both clinical and administrative variables, as well as their combination, to identify frequent admitters—prior to discharge—for pre-discharge interventions during the index (current) admission. This model has been implemented to generate daily lists of high-risk patients in all public hospitals for customized discharge procedures within 24 h of admission. Practically, we demonstrate the viability of using AI and data from electronic medical records to build a near real-time nationwide prediction tool for individual-centric discharge. Importantly, we highlight the cocktail of catalysts needed to spur successful implementation: People, processes, and change management [3,4].

We first provide a brief background of Singapore’s healthcare challenges, followed by a synthesis of the extant literature. The Singapore healthcare system was ranked the world’s most efficient in 2014 by Bloomberg, with one of the lowest healthcare costs per capita. However, Singapore’s multi-ethnic population of six million is one of the most rapidly aging in Asia, with an increasing life expectancy, a growing chronic disease burden, and a shrinking workforce. Singapore reached a demographic milestone in 2018, where the number of elders aged 65 years and older equaled that of youths 15 years and below. In addition, recent projections show that disability prevalence will grow by five-fold in 40 years [5]. This leads to justifiable concerns over the finitude of healthcare resources and the sustainability of the healthcare system, requiring initiatives to transform the existing hospital-centric model of care.

One of the main priorities of the transformation is to reduce unnecessary admissions and reliance on acute hospital care while increasing the role of primary and community care. This aligns with the recognition that inpatient care, particularly non-elective hospital readmissions, are significant cost drivers [6]. There is an increasing emphasis on targeting frequent admitters, as they account for a disproportionate share of visits and cost (10% of frequent admitters incur over 45% of costs) and contribute to the bed crunch problem in hospitals, while also suffering poorer health outcomes, psychological stress, and financial burdens [6]. In Singapore, frequent admitters are defined as patients with three or more inpatient admissions in 12 months. These are high-cost patients with high health service utilization and an average cost per patient approaching SG $30,000 (approximately US $22,000) [7]. A targeted “high risk” case management program is necessary to curb this problem [8].

The Singapore Ministry of Health (MOH) initiated a transitional care model that focused on community care for frequent admitters. However, the success of this model of care will be predicated on the MOH’s ability to identify and target high-risk patients through a nationwide risk stratification scheme [9,10,11,12]—specifically, a predictive model to identify frequent admitters prior to discharge.

There are many published tools for the early identification of high-risk patients, but they have modest predictive value, are based largely on post-discharge administrative data, or are limited to a specific clinical condition (e.g., heart failure) or subset of patients (e.g., Medicare patients in a specific state or hospital) [9,13,14,15,16]. These limitations prevent accurate and timely identification of high-risk patients amongst a more general medical inpatient population to provide actionable data for intervention prior to discharge [8,14,15,17]. Clinically actionable data need to be available early during the hospitalization to trigger effective discharge planning and to allow the care team to engage the patient during the hospitalization period.

Given the need to identify frequent admitters prior to discharge, we developed a predictive model to risk stratify patients for enrolment into the individual-centric discharge planning program across all Singapore public hospitals. This model leverages Singapore’s nationwide electronic medical records that integrate administrative and clinical data from all public hospitals.

## 2. Materials and Methods

### 2.1. Dataset and Outcomes

The cohort used to develop the model included all patients who gained admission to public hospitals within one calendar year (Year 0). These public hospitals serve a largely similar patient population in their geographic location—by design, social and housing policies in Singapore promote a mix of ethnicity and age in every town. The dataset did not include patients who were visiting Singapore as tourists or were less than 18 years of age. The inclusion criteria were Singapore citizens or residents who were admitted to any public hospital within one calendar year. The latest admission in the study period was indicated as the index admission. An extra two years of datapoints prior to Year 0, with none of the above exclusion criteria, were extracted to capture and analyze the complete clinical history of patients. This included clinical data and acute-care utilization before the index admission. The inpatient visits in the subsequent calendar year (for one calendar year after Year 0), were used to calculate the frequency of all-cause readmissions within 12 months of the index admission discharge date. The primary data source was Singapore’s nationwide electronic medical records that dynamically collected data from all public hospitals, community hospitals, and other public healthcare institutions. The ethics approval for the study was provided by the HPB Medical and Dental Board (HP24:03/31-2).

The key outcome of the predictive model was three or more non-elective inpatient admissions occurring within 12 months from index admission discharge. A total of 70 predictor variables were clustered into three primary categories: Demographics, previous healthcare utilization, and medical conditions. Demographics and patient-related data included financial status, indicated by whether the patient was eligible for the Community Health Assist Scheme (CHAS) card, a medical and dental care subsidy scheme for Singaporeans, with a monthly household income per capita of SG $1100 (circa US $820) or less. Healthcare utilization history included variables such as “cumulative length of stary (LOS) in the past 12 months,” frequency of specialist outpatient clinic visits, frequency of emergency clinic visits, and frequency of emergency admissions that were classified as unplanned admissions. Comorbidities were measured by the patient’s Charlson Comorbidity Index (CCI) five years prior to the index admission. Data from the index admission (e.g., results from inpatient laboratory tests and length of stay) were not included in our predictive model. This was due to the need to generate prediction results within 24 h of hospital admission to trigger the necessary individual-centric discharge planning.

### 2.2. Analytic Plan

We developed models using machine learning algorithms that included gradient boosting machine, random forest, lasso, and logistic regression. We included four commonly used techniques following the machine learning no-free-lunch theorem [18] that no machine learning algorithm is any more efficacious than another, thus encouraging the comparison of multiple techniques. To validate the models, the dataset was randomly split into testing (80%) and validation (20%). Ten-fold cross validation was employed on the testing set to distil the parameters and minimize over-fitting. The default hyperparameters used were from the Scikit-learn package in R (e.g., loss function set at “deviance”, learning rate set at 0.1, and subsample default of 1.0) [19,20]. The performance parameters of the predictive model were the area under curve (AUC) and sensitivity. We set the PPV at 70%, a clinical decision to better identify the appropriate group of patients for our intervention. An overly stringent PPV will lead to the identification of “very high-risk” patients instead of “high-risk” ones that are the target of our study. We validated this approach with our clinical committee to ensure it made sense from a public health system implementation perspective. Our analyses employed R version 4.0.4.

## 3. Results

In total, 183,106 patients were admitted to Singapore public hospitals within the year. The excluded patients (*N* = 32,784) did not meet the inclusion criteria. The remaining 150,322 patients were included in the analysis, of which 19,130 (12.7%) had three or more readmissions within a 12-month period. Table 1 presents the results of the machine learning algorithms conducted on the validation dataset. The 70% PPV was set to include more patients, as a higher PPV could lead to limited detection of patients at higher risk. The best performer was gradient boosting based on the AUC and sensitivity.

The strongest predictor was the cumulative length of stay (LOS) in the past 12 months. Although few studies have specifically evaluated the predictors for risk of frequent admissions, our finding is consistent with the existing literature on 30-day, 90-day, and 12-month readmission risks [6,9,12,14,15,21,22]. Interestingly, cumulative LOS was a stronger predictor than the number of previous admissions, as it is a better proxy for acute care utilization [19]. Another important predictor is the “number of days from previous non-elective admission”, which is different from most studies that included both elective and non-elective admissions. We argue that homing in on the length of time since non-elective admission will improve the predictive power of existing models. Of note, our model did not include LOS of the index admission—a key predictor in other models (e.g., LACE index and HOSPITAL score) [21,22]—since our predictive model was used to identify frequent admitters for individual-centric pre-discharge interventions during the index admission. We did not find evidence for multicollinearity, as the variance inflation factor (VIF) scores of prior statistical and predictive models were below the stringent criteria of five [23].

## 4. Discussion

This is one of the first known individual-centric discharge models implemented nationwide. Previous studies were smaller in scale—either within a hospital cluster or the models was not created for near real-time prediction. We summarize the conceptual and practical contributions.

Conceptually, our model is among a handful that uses administrative data to identify high-risk patients in near real time. Previous systematic reviews found that the number of studies using administrative data started small and has grown considerably [14]. However, their predictive abilities remain modest (AUC below 0.70) [12,13,24]. While it is not possible for a true comparison across countries due to differences in medical records and health systems, our model had a better outcome (AUC: 0.79). Importantly, our model was designed for implementation prior to discharge.

Practically, through this national initiative, we learned that the art of implementation is equally, if not more, important than the science of predictive analytics [2,25]. We distilled three learning points that draw from and contribute to managerial epidemiology [3,4]. First, change management is paramount, otherwise any predictive model, no matter how accurate, may not be accepted and implemented. Managing change does not happen after models are built, but at the ideation phase when stakeholders are engaged and consulted. At this early stage, we found it helpful to engage a multidisciplinary team to conceptualize the model blueprint and brainstorm how it could be deployed. The project team consisted of nurses, hospital operations, policymakers, IT heads, data scientists, and subject matter experts. After the initial scoping meetings, we brought in the relevant stakeholders at different parts of the value chain from ideation to implementation. Importantly, these diverse roles represented different stakeholder groups, and served as catalysts for change management throughout.

Second, business process re-engineering is necessary. The biggest risks to the success of new tools are old processes. If existing processes are incompatible with new tools, staff become confused and frustrated. At best, they revert to old ways, and at worst, they perceive AI as a “troublemaker” or conclude that AI cannot work in healthcare—shrinking the reservoir of willingness to try out future applications. In reality, it is the process that needs mending, not the AI applications, but staff conflate the two when either fail. Another driver for process re-engineering is system architecture changes, as described by Ta and colleagues [26]. Essentially, process changes need to be worked out early for successful implementation.

Third, another critical ingredient is people—specifically, ramping up the talent mix for implementation. Our model identified patients at high risk of re-admission and enrolled them in a community nursing program, prior to discharge, where healthcare professionals visited these patients in the community. A major challenge was convincing nurses, who aspired to work in hospitals, to serve in the community, where the level of perceived prestige is lower. In the community, psychosocial issues become more salient—discrimination, ageism [27,28,29,30,31], and caregiving [32]. Cultural differences that permeate daily life [33,34,35] are accentuated and may nuance participants’ responses to interventions. Nevertheless, differences matter only if they make a difference—community nurses and healthcare workers need a different set of skills and cultural aptitude to deal with risks and complexity [36,37,38,39]. In essence, human capital is an important cog in the implementation wheel; hence, it requires a fundamental re-think of personnel selection, competency frameworks, and development pathways for community nursing.

Despite our best efforts, there are limitations in our study. In the model, access to financial support is measured by a proxy measure, namely, those with a household monthly income of below SG $1100 (circa US $820) and enrolled in the Community Health Assist Scheme (CHAS) blue card scheme. As not all eligible citizens may have applied for CHAS, this proxy may not fully capture the status of patients. There are other variables that capture individual care differences and individual care processes that could not be included as they were not yet available in national medical records. This is a significant limitation that should be addressed in future studies—specifically, the inclusion of socioeconomic, mental health variables, individual care process variables. In addition, as this was a retrospective study, it is not possible to infer causal associations between the independent variables and the outcome—this was not our intention. The model’s primary role was to identify high-risk patients to be enrolled into a nationwide intervention program across 14 public hospitals serving 6 million residents.

## 5. Conclusions

Predictive analytics move the needle in healthcare. Singapore is one of the first known countries to implement a nationwide predictive model to provide all public hospitals with a daily list of high-risk patients for individual-centric discharge procedures. Predictive analytics provide a “filter” for first-level identification that was traditionally carried out by clinicians during ward assessments. With its near real-time capabilities, it dramatically increases efficiency and accuracy to enable targeted intervention with minimal clinical resources.

## Figures and Tables

**Table 1:** Performance of the models for individual-centric discharge ranked by the AUC and PPV.

Model	AUC ^1^	Sensitivity (70% PPV ^2^)
Gradient boosting machine	0.79	0.39
Random forest	0.78	0.37
Lasso	0.78	0.35
Logistic regression	0.78	0.33

^1^ AUC: Area under the curve is a criterion to distinguish the performance of machine learning models—a higher score denotes better performance. ^2^ PPV: Positive predictive value.

## Data Availability

Restrictions apply to the availability of the study’s data. Data were analyzed via limited secure access at the MOH.
